# Non-Hermitian Generalization of Rényi Entropy

**DOI:** 10.3390/e24111563

**Published:** 2022-10-30

**Authors:** Daili Li, Chao Zheng

**Affiliations:** Department of Physics, College of Science, North China University of Technology, Beijing 100144, China

**Keywords:** Rényi entropy, quantum information, non-Hermitian Hamiltonian, two-level quantum systems

## Abstract

From their conception to present times, different concepts and definitions of entropy take key roles in a variety of areas from thermodynamics to information science, and they can be applied to both classical and quantum systems. Among them is the Rényi entropy. It is able to characterize various properties of classical information with a unified concise form. We focus on the quantum counterpart, which unifies the von Neumann entropy, max- and min-entropy, collision entropy, etc. It can only be directly applied to Hermitian systems because it usually requires that the density matrices is normalized. For a non-Hermitian system, the evolved density matrix may not be normalized; i.e., the trace can be larger or less than one as the time evolution. However, it is not well-defined for the Rényi entropy with a non-normalized probability distribution relevant to the density matrix of a non-Hermitian system, especially when the trace of the non-normalized density matrix is larger than one. In this work, we investigate how to describe the Rényi entropy for non-Hermitian systems more appropriately. We obtain a concisely and generalized form of α-Rényi entropy, which we extend the unified order-α from finite positive real numbers to zero and infinity. Our generalized α-Rényi entropy can be directly calculated using both of the normalized and non-normalized density matrices so that it is able to describe non-Hermitian entropy dynamics. We illustrate the necessity of our generalization by showing the differences between ours and the conventional Rényi entropy for non-Hermitian detuning two-level systems.

## 1. Introduction

Entropy, which was proposed by Clausius [1] when he was investigating thermodynamics in the nineteenth century, has become a significant concept with different intensions in a variety of areas, such as statistical thermodynamics, classical and quantum information science, etc. Specifically, Boltzmann and Gibbs developed it and gave a statistic meaning [2] to entropy. In the twentieth century, von Neumann [3,4] and Shannon [5] independently introduced the concept of entropy and gave similar definitions in mathematics to describe quantum and classical channels in communication. Now, the concept has become a key concept and is widely applied in information theory.

In 1930s, von Neumann extended the classical concept of entropy into the quantum domain, i.e., von Neumann entropy in quantum statistical mechanics, after establishing a rigorous mathematical framework for quantum mechanics (with his work *Mathematische Grundlagen der Quantenmechanik* [3,4]). In 1940s, enlightened by the thermodynamic entropy, Shannon [5] independently gave a definition to describe classical channels in communication and decided to use the name of entropy after von Neumann’s suggestion, which is the well-known Shannon entropy in classical information theory. Besides classical and quantum statistical mechanics and information science, entropy has also become a widely used concept with different definitions and meaning in various areas, such as astrophysics, life science, etc.

Subsequently, many concepts related to information entropy are proposed and defined both in classical and quantum versions, respectively, to describe classical and quantum physical systems statistically from different aspects for applications such as relative entropy [6], Rényi entropy [7,8], Hartley entropy [9], min- and max-entropy [10,11,12], collision entropy [13,14], mutual information [15,16], etc. Ref. [17] Now, quantum entropies have been widely applied to investigate quantum systems and their characteristics [8,10,18,19] from the view of quantum information, quantifying the uncertainty of quantum measurements, quantum correlations, etc. For example, as the quantum counterpart of Shannon entropy, von Neumann entropy [3] has been naturally and widely used in quantum information science as a measurement of the uncertainty of information sources in quantum systems and channels. Although quantum mechanics was born with a Hermitian formalism to conserve the normalized probability, non-Hermitian (NH) quantum physics [20,21,22,23,24,25,26,27,28,29,30,31,32,33,34,35,36,37,38,39,40,41,42,43,44,45,46] attracted increasingly interesting research in that they are closely related to open and dissipative systems [47,48,49,50,51,52,53,54,55], which are normal in the real world and have many applications [56,57,58,59,60,61,62,63,64,65,66,67,68,69]. Few studies of entropy emphasize non-Hermitian (NH) systems and dynamics, while most settings of quantum entropy is for Hermitian cases. Sergi and coworkers introduced non-Hermitian generalizations of von Neumann entropy and linear entropy [70,71,72,73,74]. Quantum Rényi entropy unifies the von Neumann entropy and many other quantum measures settings such as collision entropy and Renner’s max- and min-entropy, and can be applied in different theoretical tasks with one concise form [7]. For example, quantum Rényi entropy is able to characterize the error exponents and strong converse exponents for quantum hypothesis testing and quantum communication theory [75,76,77,78,79,80,81]. However, there is no definition of quantum Rényi entropy for general non-Hermitian quantum systems of which the total probability of quantum states is no longer conserved at greater than or less than 1.

In this work, we investigate the Rényi quantum entropy and generalize it to a concise generalized form that is applicable to both the Hermitian and NH cases, i.e., the trace of the density matrix can be equal to, larger than, or less than one. Our new definition also extends the order α of the Rényi entropy, combining the Hartley entropy (α=0) and the original Rényi entropy (α>0) that includes the max-entropy (0<α<1), von Neumann entropy (α=1), min-entropy (α=+∞), and other positive real numbers, which can be applied to computer science and classical and quantum information theories.

This paper is structured as follows. In Section 2, we introduce the α-Rényi entropy with classical setting and Hermitian quantum setting. Specifically, we briefly review the Shannon entropy and classic Rényi entropy, the von Neumann entropy and quantum Rényi entropy, and the NH-generalization of von Neumann entropy. In Section 3, we give the definition of NH quantum α-Rényi entropy and its relevant extensions. Specifically, we give the NH Rényi entropy definition and explore the relation with Hermitian counterpart in Section 3.1 and demonstrate the necessity of our generalization in Section 3.2. In Section 4, we illustrate the necessity of our generalized NH Rényi entropy by tunneling models, which shows that our generalized Rényi entropy is more appropriate than the original one when characterizing NH systems. In Section 5, we give discussions and conclusions.

## 2. α-Rényi Entropy

In order to illustrate the NH generalization of Rényi entropy, we briefly review the entropy of classical and quantum systems in this section.

### 2.1. Shannon Entropy and Classic Rényi Entropy

Classical α-Rényi entropy is proposed by Alfréd Rényi in 1961, which can characterize many different information tasks by one setting [7]. Take a discrete probability distribution $P_{x}=\left\{p_{1},\ldots ,p_{n}\right\}$ over a set *X* as an example. The α-Rényi entropy is defined as follows:

(1) $$S_{\alpha }{\left(X\right)}_{P}:=\frac{1}{1-\alpha }\ln\sum_{i=1}^{n}p_{i}^{\alpha },\mathrm{where}\;\alpha \in \left(0,1\right)\cup \left(1,\infty \right).$$

When α=1, Rényi entropy becomes a Shannon entropy:

(2) $$S_{1}{\left(X\right)}_{P}=\lim_{\alpha \to 1}S_{\alpha }{\left(X\right)}_{P}=-\sum_{i=1}^{n}p_{i}\ln p_{i},$$

which can still be defined as stated in Equation (1) in the sense of taking the limit as α proceeds to one, and it is calculated by the L’Hôpital’s rule. In particular, Rényi entropy becomes Hartley entropy and min-entropy when α approaches 0 and *∞*, respectively, which have applications in cryptography. The min-entropy of a discrete probability distribution is the negative logarithm of the probability of the most likely outcome. It is not greater than the original one defined by Rényi in Equation (1) with the same probability distribution. $S_{2}{\left(X\right)}_{P}$ is called collision entropy when the order is α=2.

### 2.2. Von Neumann Entropy and Quantum Rényi Entropy

Von Neumann entropy is introduced to describe the uncertainty of quantum systems, and the definition is as follows:

(3) $$S_{1}^{H}\left(\rho \right)=-Tr\left(\rho \ln\rho \right)=-\sum_{i=1}^{n}\lambda_{i}\ln\lambda_{i},$$

where ρ is the density matrix of a quantum system, *n* is the dimension of the Hilbert space relevant to the system, and $\lambda_{i}$ (i=1,…,n) are the eigenvalues of ρ.

Similarly to von Neumann and Shannon entropies, Rényi entropy is generalized to a Hermitian quantum version [79]. This version of quantum Rényi entropy requires the trace of density matrix to satisfy Tr(ρ)∈(0,1] depending on incomplete and complete probability distributions. It is always applied to conventional Hermitian quantum systems, of which the traces of the density matrices are conserved to one by the Hermiticity, so we call it the Hermitian quantum Rényi entropy. For a density matrix ρ with eigenvalues $\lambda_{1}\geq \ldots \geq \lambda_{n}$, the Hermitian quantum α-Rényi entropy is defined as follows:

(4) $$S_{\alpha }^{H}\left(\rho \right)=\frac{\ln Tr\rho^{\alpha }}{1-\alpha }=\frac{\ln\sum_{i=1}^{n}\lambda_{i}^{\alpha }}{1-\alpha },$$

where α∈(0,1)∪(1,∞) and Tr(ρ)=1. Similarly to the classical α-Rényi entropy, Hermitian quantum α-Rényi entropy becomes von Neumann entropy in Equation (3) when α approaches one, i.e., $S_{1}^{H}\left(\rho \right)=\lim_{\alpha \to 1}S_{\alpha }^{H}\left(\rho \right)$. The order α of Rényi entropy can be extended to zero and *∞* by taking the relevant limits in Equation (4). Renner and Kônig [10,19] further unified two cases when α approached zero and *∞* into the quantum Rényi entropy in Equation (4), which are the smooth min-entropy and max-entropy, respectively:

(5) $$\left\{\begin{array}{cc} S_{max}=S_{0}^{H}\left(\rho \right) & =\ln n,\\ S_{min}=S_{\infty }^{H}\left(\rho \right) & =-\ln(\max_{i\in [i,n]}\lambda_{i}). \end{array}\right.$$

where $S_{0}^{H}\left(\rho \right)$ is called the quantum Hartley (or max-) entropy that measures the size of the probability space, $S_{\infty }^{H}\left(\rho \right)$ is called the quantum min-entropy that is the minus times the logarithm of the maximum of its operator spectrum. Therefore, the order α of Hermitian quantum Rényi entropy is extended to [0,∞]. When α varies from zero to infinity, $S_{\alpha }^{H}\left(\rho \right)$ monotonically decreases [18]. In non-asymptotic theory, Renner and Wolf show that smoothing α-Rényi entropy can be separated into three classes, i.e., α<1, α=1, and α>1 [8,18,80,81]. Renner then chooses the min-entropy $S_{min}$ as the representative of the smooth Rényi entropy when the order is α>1 [8,12]. Two max-entropies, $S_{0}^{H}\left(\rho \right)$ or $S_{\frac{1}{2}}^{H}\left(\rho \right)$, can be chosen as representatives of smooth Rényi entropy when the order is α<1 [8]. The choice of $\frac{1}{2}$ is motivated by the duality relation of the conditional max-entropy [12]. Here, we provide the form of the max-entropy with the order $\alpha =\frac{1}{2}$.

(6) $$S_{\frac{1}{2}}^{H}\left(\rho \right)=2\ln Tr\left(\rho^{\frac{1}{2}}\right).$$

For α=2, $S_{2}^{H}\left(\rho \right)$ is the collision entropy of quantum systems [19]. This quantity is widely used in quantum information processing and quantum cryptography.

The previous discussion of entropies is for a Hermitian quantum system, of which density matrix ρ is always normalized. However, the density matrix Ω of a non-Hermitian system is non-normalized, of which the trace can be either less or larger than one. Therefore, the previous definition of Rényi entropy is not applicable to NH systems. Although we can normalize the non-normalized density matrix Ω of an NH system to a normalized one as follows

(7) $$\rho =\frac{\Omega }{Tr(\Omega )},$$

it is not appropriate to use the previous definitions of entropies of Hermitian systems to describe NH systems [73,74]. The reason is that, for quantum systems, the normalized density matrix ρ maintains a probability interpretation of operators under non-Hermitian dynamics, while the density matrix, Ω, appropriately describes the gain or loss of probability associated with the coupling to sinks or sources [73,74]. Therefore, Ω and ρ are both key factors that require consideration to describe the quantum information in non-Hermitian systems.

### 2.3. The Non-Hermitian Generalization of Von Neumann Entropy

One first attempt to develop the entropy relative to NH systems was made by Sergi et al. [73], in which they generalized the von Neumann entropy defined for Hermitian quantum systems to its NH counterpart $S_{NH}$. In their work, the von Neumann NH entropy is defined by using both the normalized density matrix (ρ) and non-normalized density matrix (Ω) [73]:

(8) $$S_{NH}:=-Tr\left(\rho \ln\Omega \right),$$

where we set the Boltzmann constant to be 1. Compared with the von Neumann entropy, $S_{NH}$ can properly describe the irreversible process in non-Hermitian cases, and it can monitor the onset of the disorder in quantum dissipative systems. Sergi and Zloshchastiev [73] illustrate the NH entropy $S_{NH}$ by the two-level tunneling models with non-Hermitian detuning, in which $S_{NH}$ changes while the von Neumann entropy does not.

## 3. Our NH Quantum α-Rényi Entropy

We propose a generalized quantum Rényi entropy for NH systems and illustrate the necessity and properties in this section.

### 3.1. NH Rényi Definition

To describe an NH system properly, our NH α-Rényi entropy is defined using both the non-normalization density matrix Ω and the normalization one ρ as follows.

(9) $$S_{\alpha }\left(\Omega \right):=\left\{\begin{array}{cc} \frac{\ln\;Tr(\Omega^{\alpha -1}\rho )}{1-\alpha }=\frac{\ln\;\sum_{i=1}^{n}\lambda_{i}^{\alpha }{\left(Tr\Omega \right)}^{\alpha -1}}{1-\alpha }, & \alpha \in (0,1)\cup (1,\infty ),\\ S_{0,1,\infty }\left(\Omega \right)=S_{\alpha \to 0,1,\infty }\left(\Omega \right), & \alpha =0,\;1,\;\infty . \end{array}\right.$$

We set $S_{1}\left(\Omega \right)$ as $S_{\alpha \to 1}\left(\Omega \right)$, i.e., the limitation of the first line in Equation (9) when α approaches 1. The relation between the Hermitian and NH quantum α-Rényi entropies is described as follows.

(10) $$S_{\alpha }\left(\Omega \right)=S_{\alpha }^{H}\left(\rho \right)-\ln Tr\Omega .$$

It indicates that the deviation of TrΩ from unity can be obtained by measuring the difference of $S_{\alpha }\left(\Omega \right)$ and $S_{\alpha }^{H}\left(\rho \right)$. Because the second term in Equation (10), lnTrΩ, does not depend on α, our non-Hermitian α-Rényi entropy has the same monotonicity with $S_{\alpha }^{H}\left(\rho \right)$, which can be proved as $S_{\alpha }\left(\Omega \right)$ monotonically decreases as order α increases from Equation (10).

### 3.2. Typical Cases of Our NH Quantum α-Rényi Entropy with Fixed α

We specify the order α to be (i) 1, (ii) 0, (iii) +∞, (iv) $\frac{1}{2},$ and (v) 2, investigating the properties and discussing the significance of the relevant α-Rényi entropy.

(i) NH von Nuemann entropy (α=1)

Using L’Höpital’s rule, we can derive the following for α = 1 in Equation (9).

(11) $$S_{1}\left(\Omega \right)=\lim_{\alpha \to 1}S_{\alpha }\left(\Omega \right)=\lim_{\alpha \to 1}\frac{{\left(Tr\Omega^{\alpha -1}\rho \right)}^{\prime }}{-Tr(\Omega^{\alpha -1}\rho )}=\lim_{\alpha \to 1}\frac{Tr(\Omega^{\alpha -1}\rho \ln\Omega )}{-Tr(\Omega^{\alpha -1}\rho )}=-Tr\left(\rho \ln\Omega \right),$$

Comparing Equation (11) with Equation (8), the generalized NH von Neumann entropy [73] $S_{NH}$ in Equation (8) is obtained by our NH Rényi entropy when α=1, i.e., $S_{1}=S_{NH}$.

(ii) NH max-entropy (α=0)

We extend the order α to 0 here. We define and derive the NH max-entropy for NH quantum systems as follows (referring to Appendix A.1):

(12) $$S_{0}\left(\Omega \right):=\lim_{\alpha \to 0}S_{\alpha }\left(\Omega \right)=\ln n-\ln Tr\Omega ,$$

which can be seen as the NH quantum version of Hartley entropy.

(iii) NH min-entropy (α=∞)

The order α in Equation (9) can be extended to *∞* and is defined as follows:

(13) $$S_{\infty }\left(\Omega \right):=-\ln\left(\max_{1\leq i\leq n}\left(\lambda_{i}\right)\right)-\ln\left(Tr\Omega \right),$$

which can refer to Appendix A.2. $S_{\infty }\left(\Omega \right)$ is the NH quantum version of the min-entropy.

(iv) NH max-entropy ($\alpha =\frac{1}{2}$)

Motivated by the duality relation of the conditional entropies [8,12], $S_{\frac{1}{2}}\left(\Omega \right)$ can be seen as the definition of NH quantum max-entropy.

(14) $$S_{\frac{1}{2}}\left(\Omega \right)=2\ln Tr\left(\Omega^{-\frac{1}{2}}\rho \right).$$

(v) NH collision entropy (α=2)

The collision entropy for NH quantum system can be calculated by the following.

(15) $$S_{2}\left(\Omega \right)=-\ln Tr\left(\Omega^{-1}\rho \right).$$

## 4. Illustration of the Necessity of Our Generalized NH Quantum Rényi Entropy

From the above sections, the conventional definition of Rényi entropy includes the normalized density matrix ρ of which the trace is conserved to one, and it is always used to deal with a Hermitian quantum system. However, the density matrix Ω of an NH quantum system is non-normalized, and it reflects the degree of non-Hermiticity in the deviation of the trace of Ω from one (greater or less). Therefore, it is rational to consider both the normalized density matrix ρ and the non-normalized density matrix Ω, when defining and calculating the Rényi entropy for NH systems. Our NH quantum α-Rényi entropy can properly describe the information interaction between the system and the bath than the original Rényi entropy. We will illustrate the necessity of our NH entropy by some models in this section.

For a two-dimensional quantum system, the Hamiltonian is set as follows:

(16) $$H_{a}=H-i\Gamma ,$$

where $H=\frac{1}{2}\left(H_{a}+H_{a}^{†}\right)$ and $\Gamma =\frac{i}{2}\left(H_{a}-H_{a}^{†}\right)$ are Hermitian [70]. $H_{a}$ can be typical NH two-level systems, such as PT-symmetric and P-pseudo-Hermitian systems. Equation (16) is also closely related to the complex scaling method (CSM) [20,21,22,23,24,25,26,27] of mathematical physics, since it is similar to the non-stationary states of nuclear physics in Gamow’s theory [20].

According to quantum Liouville equation [28], the evolution equation for the non-normalized density Ω and normalized density ρ can be written as follows.

(17) $$\begin{array}{cc} \frac{d}{dt}\Omega = & -\frac{i}{\hbar }\left(H\Omega -\Omega H\right)-\frac{1}{\hbar }\left(\Gamma \Omega +\Omega \Gamma \right),\\ \frac{d}{dt}\rho = & -\frac{i}{\hbar }\left(H\rho -\rho H\right)-\frac{1}{\hbar }\left(\Gamma \rho +\rho \Gamma \right)+\frac{2}{\hbar }\rho Tr\left(\rho \Gamma \right). \end{array}$$

Notice that the trace of ρ is conservative, while the trace of Ω can be less, equal, or greater than one during the evolution with the NH Hamiltonian. The original subsystem, which is illustrated by the Hermitian *H* with the environment effect Γ, can be effectively described by the evolution for non-normalized density Ω from the theory of open systems [47,71], while the evolution for normalized density ρ effectively describes both the original subsystem illustrated with *H* with the environment effect Γ and the additional term restoring the conservation of the overall probability.

Let us consider a ‘gauge’ transformation shifting the eigenvalue of the original one to the following:

(18) $$H_{a}\to H_{a}+c_{0}I,$$

where $c_{0}$ can be an arbitrary complex number, and *I* is the identity matrix. Under this ‘gauge’ shift, the normalized density matrix ρ is invariable, while the non-normalized density operator Ω will be changed. If $c_{0}$ is set as a pure imaginary number, then the following relation between ρ and Ω can be obtained [73]:

(19) $$\begin{array}{cc} \rho^{\prime } & =\rho ,\\ \Omega^{\prime } & =\Omega e^{-\frac{2ic_{0}t}{\hbar }}, \end{array}$$

where $\rho^{\prime }$ and $\Omega^{\prime }$ are density matrices after the ‘gauge’ shift. Consequently, our NH α-Rényi entropy $S_{\alpha }\left(\Omega \right)$ will be affected by the ‘gauge’ transformation, while the conventional one $S_{\alpha }^{H}\left(\rho \right)$ is ‘gauge’ invariant. It is rational that the energy shift should affect the entropy of NH systems. Our generalized NH Rényi entropy is more appropriate for describing NH quantum systems, since it is able to reflect the change induced by changes in NH Hamiltonians.

We now illustrate our α-Rényi entropy by using two-level tunneling models. By setting $H=-\hbar \Delta \sigma_{x}$ and $\Gamma =\hbar \gamma \sigma_{z}$ in Equation (16), the Hamiltonian is deescribed as follows:

(20) $$H_{a0}=-\hbar \Delta \sigma_{x}-i\hbar \gamma \sigma_{z},$$

where parameters Δ (>0) and γ (>Δ) are real; $\sigma_{x}=\left(\begin{array}{cc} 0 & 1\\ 1 & 0 \end{array}\right)$ and $\sigma_{z}=\left(\begin{array}{cc} 1 & 0\\ 0 & -1 \end{array}\right)$ are Pauli matrices. After the ‘gauge’ shift in Equation (18), $H_{a0}$ is transformed to $H_{ak}$:

(21) $$H_{a0}\to H_{ak}=H-i\Gamma +c_{0}I=-\hbar \Delta \sigma_{x}-i\hbar \gamma \sigma_{z}-\frac{1}{2}ik\hbar \Delta \mu I,$$

where *k* is a positive real number that indexes a type of ‘gauge’ shift, and $\mu =\sqrt{{\left(\frac{\gamma }{\Delta }\right)}^{2}-1}$. From Equations (4) and (19), the normalized density matrix ρ and the conventional quantum α-Rényi entropy are invariant under the ‘gauge’ shift, whereas our NH α-Rényi entropy is changed because the non-normalized density matrix Ω is changed by the ‘gauge’ shift.

In the following subsections, we will investigate the NH quantum α-Rényi entropy dynamics of the time-evolutions of the two-level tunneling models with different parameter *k* based on the non-normalized density matrix in Equation (21). We find that these models with different *k* in Equation (21) have the same $S_{\alpha }^{H}\left(\rho \right)$, while there are three distinguishable patterns of our NH α-Rényi entropy $S_{\alpha }\left(\Omega \right)$ depending on parameter k∈[0,+∞) at a large time after the evolutions of the NH two-level tunneling models in Equation (21):

(22) $$S_{\alpha }{\left(\Omega \right)}_{t\to \infty }\propto \left(k-2\right)\mu t\Delta ,$$

i.e., (1) 0≤k<2, $S_{\alpha }\left(\Omega \right)\propto -\;t$ (decreasing), (2) k=2, $S_{\alpha }\left(\Omega \right)\to$ a constant, or (3) k>2, $S_{\alpha }\left(\Omega \right)\propto +\;t$ (increasing). We illustrate these three patterns in the following three subsections, setting k=0, 2, and 3. More details are shown in Appendix B.

We first investigate $S_{\alpha }^{H}\left(\rho \right)$ of the NH two-level tunneling models in Equation (21). We initialize the system to a mixed state:

(23) $$\rho \left(0\right)=\Omega \left(0\right)=\left(\begin{array}{cc} p & 0\\ 0 & 1-p \end{array}\right),$$

where p∈[0,1] and it is real. Notice that when p=0 or 1, the initial state is pure. Unless otherwise specified, we use the state in Equation (23) as the default initial state in most of the following situations. By solving the Schödinger equation, we can obtain the evolution operator, $e^{-i\frac{t}{\hbar }H_{a0}}$, and we obtain the evolved density matrix:

(24) $$\Omega =f_{y}\left(t\right)\sigma_{y}+f_{z}\left(t\right)\sigma_{z}+F\left(t\right)I,$$

where $f_{y}\left(t\right)=x_{2}x_{3}+\bar{p}x_{1}x_{3},f_{z}\left(t\right)=\frac{1}{2}\bar{p}\left(x_{1}^{2}+x_{2}^{2}-x_{3}^{2}\right)+x_{1}x_{2}$, $F\left(t\right)=\frac{1}{2}\left(x_{1}^{2}+x_{2}^{2}+x_{3}^{2}\right)-\bar{p}x_{1}x_{2}$, $\tau =t\Delta ,\;\bar{p}=2p-1,\;x_{1}=\cos\left(i\mu \tau \right),\;x_{2}=\frac{\gamma }{i\mu \Delta }\sin\left(i\mu \tau \right)$ and $x_{3}=\frac{1}{i\mu }\sin\left(i\mu \tau \right)$. We then derive the eigenvalues of Ω.

(25) $$\lambda_{1}=F\left(t\right)-\sqrt{{f_{x}\left(t\right)}^{2}+{f_{y}\left(t\right)}^{2}+{f_{z}\left(t\right)}^{2}},\;\;\lambda_{2}=F\left(t\right)+\sqrt{{f_{x}\left(t\right)}^{2}+{f_{y}\left(t\right)}^{2}+{f_{z}\left(t\right)}^{2}}.$$

**Figure 1:**
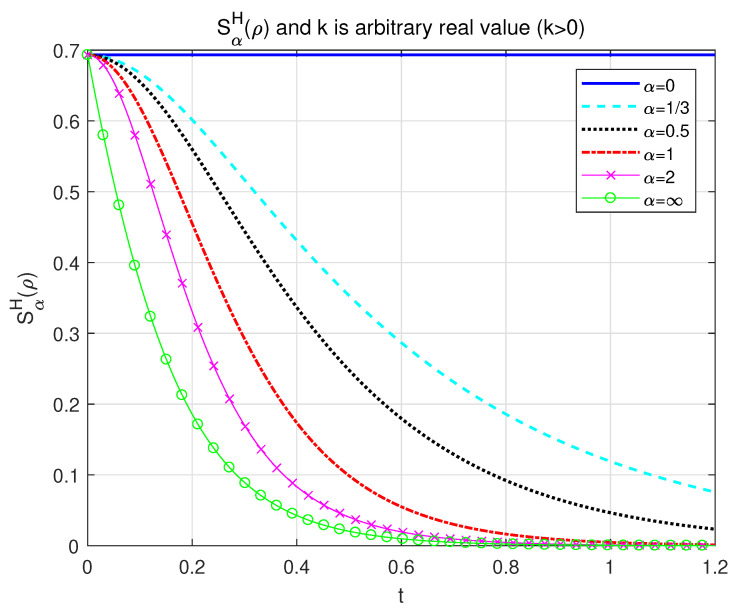
The dynamics of the conventional α-Rényi entropy as a function with respect to time *t*. *p* is set to be $\frac{1}{2}$ for different α=0 (solid line), $\frac{1}{3}$ (long-dashed line), $\frac{1}{2}$ (dotted line), 1 (dashed-dotted line), 2 (line with marker ‘x’), and *∞* (line with marker ‘o’).

Therefore, the normalized density matrix is as follows:

(26) $$\rho =\frac{f_{x}\left(t\right)}{2F(t)}\sigma_{x}+\frac{f_{y}\left(t\right)}{2F(t)}\sigma_{y}+\frac{f_{z}\left(t\right)}{2F(t)}\sigma_{z}+\frac{1}{2}I,$$

and the conventional quantum α-Rényi entropy in Equation (4) is as follows:

(27) $$S_{\alpha }^{H}\left(\rho \right)=\frac{1}{1-\alpha }\ln\left(\lambda_{1}^{\alpha }+\lambda_{2}^{\alpha }\right)-\frac{\alpha }{1-\alpha },\ln\left(2F\left(t\right)\right),$$

which is a function of time *t* shown in Figure 1 and is limited to zero at large time periods.

### 4.1. A Two-Level Tunneling Model with Non-Hermitian Detuning and Decreasing NH Entropy

If we set *k* to be 0 for $H_{ak}$ in Equation (21), the Hamiltonian becomes $H_{a0}$ as that in Equation (20), which is both PT-symmetric and P-pseudo-Hermitian. The NH α-Rényi entropy that we define in Equation (9) as a function of time *t* during the system’s evolution from the initial state in Equation (23) can be calculated as follows.

(28) $$S_{\alpha }\left(\Omega \right)=\frac{1}{1-\alpha }\ln\left(\lambda_{1}^{\alpha }+\lambda_{2}^{\alpha }\right)-\frac{1}{1-\alpha }\ln\left(2F\left(t\right)\right).$$

**Figure 2:**
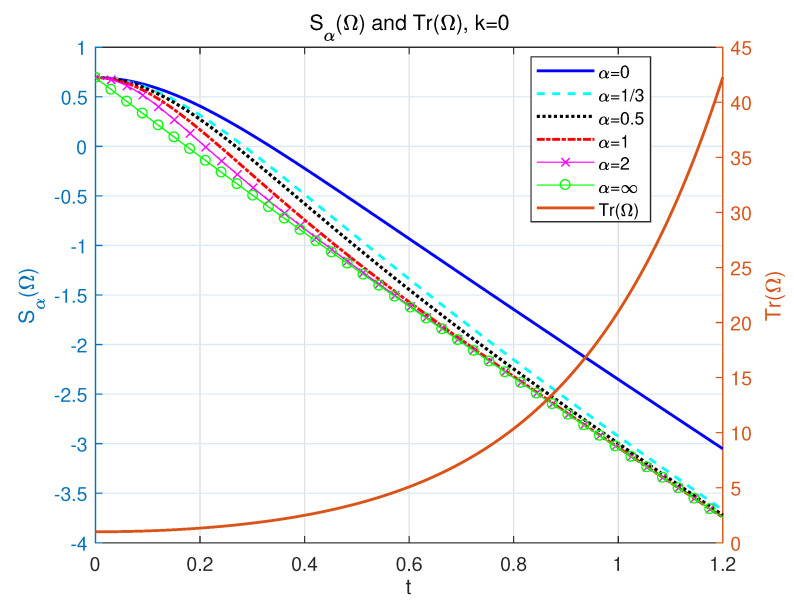
The left side of longitudinal axis: the dynamics of our NH α-Rényi entropy as a function with respect to time *t*. $p=\frac{1}{2},\;k=0,\;\Delta =1$, and γ=2 for different α= 0 (solid line), $\frac{1}{3}$ (dashed line), $\frac{1}{2}$ (dotted line), 1 (dashed-dotted line), 2 (line with marker ‘x’), and *∞* (line with marker ‘o’). The right side of the longitudinal axis: the profile of Tr(Ω) as a function of time *t* for the value k=0, Δ=1, and γ=2.

It is in accordance with the relation in Equation (10). By mathematical analyses, the NH α-Rényi entropy has asymptotical properties at large times, which is related to F(t) only:

(29) $$\lim_{t\to \infty }S_{\alpha }\left(\Omega \right)=-\lim_{t\to \infty }\ln\left(2F\left(t\right)\right)\propto -2\mu \tau ,$$

and it is referred to in Figure 2. The entropy becomes negative due to the change of Tr(Ω) at large time periods, which is reasonable because an NH system is relevant to open or dissipative systems. Tr(Ω) shows that the probability varies, indicating how the information flow rate of the probability flows into or out of the NH system.

### 4.2. A Two-Level Tunneling Model with Non-Hermitian Detuning (k=2) and Asymptotically Constant NH Entropy

In the previous subsection, the two-level tunneling model (k=0) exhibits that the NH entropy decreases from positive to negative as the increasing time. Here, we set the models with k=2, and it is equivalent to adding a constant decay operator to the Γ operator in Equation (20).

(30) $$H_{a2}=-\hbar \Delta \sigma_{x}-i\hbar \gamma \sigma_{z}-i\hbar \Delta \mu I.$$

By the ‘gauge’ shift in Equation (19), it is not difficult to find that both normalized density ρ and conventional α-Rényi entropy are the same as that in Equations (26) and (27). However, the non-normalized density Ω is changed by multiplying a factor.

(31) $$\Omega =e^{-2\mu \tau }\left(f_{x}\left(t\right)\sigma_{x}+f_{y}\left(t\right)\sigma_{y}+f_{z}\left(t\right)\sigma_{z}+F\left(t\right)I\right).$$

**Figure 3:**
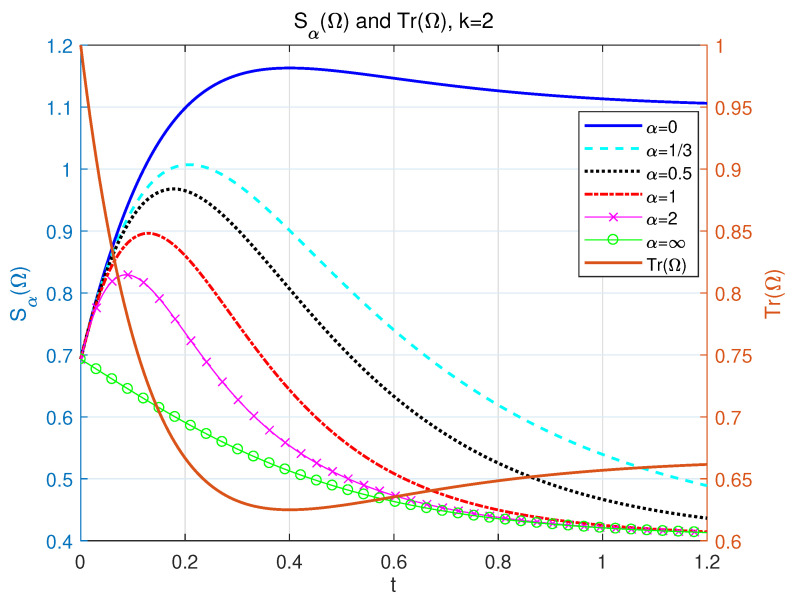
The left side of longitudinal axis: the dynamics of our NH α-Rényi entropy as a function with respect to time *t*. $p=\frac{1}{2},\;k=2,\;\Delta =1$ and γ=2 for different α = 0 (solid line), $\frac{1}{3}$ (dash line), $\frac{1}{2}$ (dotted line), 1 (dash-dotted line), 2 (line with marker ‘x’), and *∞* (line with marker ‘o’). The right side of longitudinal axis: the profile of Tr(Ω) as a function of time *t* for the value k=2, Δ=1 and γ=2.

Then, we derive our NH α-Rényi entropy by substituting Equation (31) into Equation (9):

(32) $$S_{\alpha }\left(\Omega \right)=S_{\alpha }^{H}\left(\rho \right)-\ln2F\left(t\right)+2\mu \tau ,$$

where $S_{\alpha }^{H}\left(\rho \right)$ is given by Equation (26). We calculate the asymptotical value at large time periods:

(33) $$\lim_{t\to \infty }S_{\alpha }\left(\Omega \right)=\left\{\begin{array}{c} \alpha =0,\;2\ln2-\ln\left(1+\frac{1}{\mu^{2}}-\frac{\bar{p}\gamma }{\mu \Delta }\right)\\ \alpha >0,\;\ln2-\ln\left(1+\frac{1}{\mu^{2}}-\frac{\bar{p}\gamma }{\mu \Delta }\right), \end{array}\right.$$

of which the difference from when k=0 is that the $S_{\alpha }\left(\Omega \right)$ is bounded here. The entropy will tend to a constant value at large time. Actually, k=2 is a threshold for deciding whether $S_{\alpha }\left(\Omega \right)$ will asymptotically decrease or increase at large time periods. Specifically, entropy will decrease when 0≤k<2 and increase when k>2 (discussed in the next subsection), referring to details in Appendix B. The outlines of $S_{\alpha }\left(\Omega \right)$ when $\alpha =0,\frac{1}{3},\frac{1}{2},1,2$ and *∞* are shown in Figure 3. We find two asymptotic lines in Figure 3 for α=0 and α>0, which are in accordance with the analytic results in Equation (33).

### 4.3. A Two-Level Tunneling Model with Non-Hermitian Detuning (k=3) and Increasing NH Entropy

If we set k=3, the Hamiltonian in Equation (21) becomes the following.

(34) $$H_{a3}=-\hbar \Delta \sigma_{x}-i\hbar \gamma \sigma_{z}-\frac{3}{2}i\hbar \Delta \mu I.$$

The non-normalized density Ω acquires an exponent factor.

(35) $$\Omega =e^{-3\mu \tau }\left(f_{x}\left(t\right)\sigma_{x}+f_{y}\left(t\right)\sigma_{y}+f_{z}\left(t\right)\sigma_{z}+F\left(t\right)I\right).$$

Substituting Equation (35) into Equation (10), we obtain the following.

(36) $$S_{\alpha }\left(\Omega \right)=S_{\alpha }^{H}\left(\rho \right)-\ln2F\left(t\right)+3\mu \tau .$$

By mathematical analysis, the NH α-Rényi entropy has asymptotical properties at large time periods.

(37) $$\lim_{t\to \infty }S_{\alpha }\left(\Omega \right)=\lim_{t\to \infty }\left(-\ln2F\left(t\right)+3\mu \tau \right)\propto \mu \tau .$$

**Figure 4:**
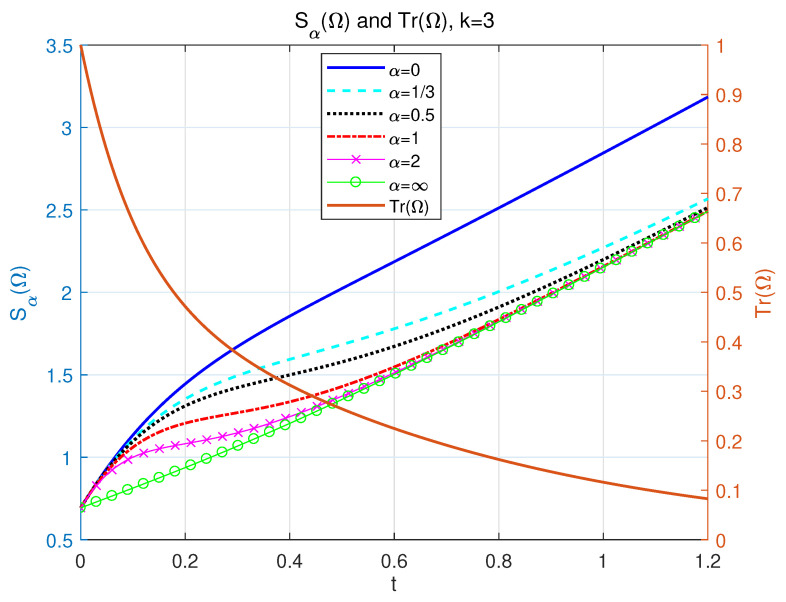
The left side of longitudinal axis: the dynamics of our NH α-Rényi entropy as a function with respect to time *t*. $p=\frac{1}{2},\;k=3,\;\Delta =1$ and γ=2 for different α = 0 (solid line), $\frac{1}{3}$ (dash line), $\frac{1}{2}$ (dotted line), 1 (dash-dotted line), 2 (line with marker “x”) and *∞* (line with marker “o”). The right side of longitudinal axis: the profile of Tr(Ω) as a function of time *t* for the value k=3, Δ=1, and γ=2.

So $S_{\alpha }\left(\Omega \right)$ will be limited to an increasing linear function at large time periods. Different α-Rényi entropies as functions of time *t* are shown in Figure 4 with $\alpha =0,\frac{1}{3},\frac{1}{2},1,2$, and *∞*.

## 5. Discussion and Conclusions

We propose a generalized α-Rényi entropy for non-Hermitian quantum systems depending both on the normalized and non-normalized density matrices. The normalized one is bounded and shows statistical averages of the the local probability distribution and the local disorder of the NH system, while the non-normalized one shows (1) the gain or loss of probability, (2) how the information flows between the system and the bath, and (3) some important features of the gain or decay process, such as the non-conservation of probability in the open system and how it is obtained from or leaks into the surrounding environment. Therefore, our generalized NH α-Rényi entropy $S_{\alpha }\left(\Omega \right)$ can signal the expected thermodynamic behavior of an open system, and it is more suitable to describe NH physical behaviors and disorder in NH systems than the conventional definition that is decided only by the normalized density matrix. Both the ’local’ disorder of the NH system and the overall probability distribution between the NH system and the environment are considered and combined into the definition of our NH entropies. Notice that our generalized NH α-Rényi entropy is compatible with the conventional α-Rényi entropy defined for classical and Hermitian quantum systems, and it becomes Sergi et al.’s generalized NH von Neumann entropy when α=1. We extend the order α of our generalized Rényi entropy to zero (generalized Hartley entropy) and the positive infinity (generalized min-entropy), i.e., our α∈[0,+∞). Thus, our generalized Rényi entropy is able to quantify NH systems from different aspects by one unified definition.

We illustrate our NH α-Rényi entropy by two-level tunneling models with different non-Hermitian detuning (i.e., the ‘gauge’ transformation). Without a loss of generality, we chose the order $\alpha =0,\frac{1}{3},\frac{1}{2},1,2$, and *∞* in each model. By mathematical analyses, the curves of our NH α-Rényi entropy with respect to time *t* became lower as α increases, while both the normalized density matrix and the conventional Rényi entropy did not change with respect to the parameter *k*. For the monotonic behavior of our NH α-Rényi entropy at large time, we found a critical value (or a threshold) k=2 of non-Hermitian detuning, at which there exists a constant limitation for our NH α-Rényi entropy. This indicates that a balance is found between the NH open system and the environment. For the NH models with the detuning parameter 0≤k<2 and k>2, our NH α-Rényi entropy will be proportional to −t in a decreasing manner and *t* in an increasing manner. These two cases indicate that there are continuous entropy flows from (or into) the NH open system into (or from) the environment. At large time periods, monotonicities of NH α-Renyi entropy and Tr(Ω) are opposite from one another, and it will be positive if Tr(Ω)<1 and negative if Tr(Ω)>1. This reflects that the flow of the probability distribution between the NH system and the bath (reflected by Tr(Ω)) governs the NH entropies more than the local entropy of the NH system (reflected by ${S_{\left(\rho \right)}}^{H}$) at large *t*. In particular, for $S_{0}\left(\Omega \right)$, the monotonicity of NH α-Renyi entropy and Tr(Ω) is opposite all the time, which means that $S_{0}\left(\Omega \right)$ is able to indicate the probability of flowing into (Tr(Ω) increasing) or out of (Tr(Ω) decreasing) the NH system from or into the bath. Hence, one can say that $S_{\alpha }\left(\Omega \right)$ describes the flow of information between the system and the bath. Sometimes, the local entropy of the NH system contributes more to NH entropies. This can be seen from the fact that there exists a short period of time in which all quantities such as $S_{\alpha }\left(\Omega \right)$, $S_{\alpha }^{H}\left(\rho \right)$, and Tr(Ω) decrease.

For the outlook, on the one hand, it is interesting to investigate how we can measure our generalized α-Rényi entropy experimentally. On the other hand, our generalization can be extended to investigate other quantities of physics and information science for NH systems, such as quantum divergences that involve two quantum density matrices.

## Appendix A. Our NH α-Rényi Entropy When α = 0 and ∞

### Appendix A.1. Max-Entropy (α = 0)

Here, we prove a special case in Equation (9) that α=0.

(A1) $$S_{0}\left(\Omega \right)=\lim_{\alpha \to 0}Tr\left(\Omega^{-1}\rho \right)=\ln Tr\left(\frac{\rho^{-1}}{Tr\Omega }\rho \right)=\ln n-\ln Tr\Omega .$$

### Appendix A.2. Min-Entropy (α = ∞)

Here, we prove a special case in Equation (9) that α=∞.

(A2) $$\begin{array}{cc} S_{\infty }\left(\Omega \right) & =\lim_{\alpha \to \infty }S_{\alpha }\left(\Omega \right)\\ \; & =\lim_{\alpha \to \infty }\frac{\ln\sum_{i=1}^{n}\lambda_{i}^{\alpha }{\left(Tr\Omega \right)}^{\alpha -1}}{1-\alpha }\\ \; & =\lim_{\alpha \to \infty }\frac{\left(\alpha -1\right)\ln\left(Tr\Omega \right)+\ln\sum_{i=1}^{n}\lambda_{i}^{\alpha }}{1-\alpha }\\ \; & =-\ln\left(Tr\Omega \right)+\lim_{\alpha \to \infty }\frac{\ln\sum_{i=1}^{n}\lambda_{i}^{\alpha }}{1-\alpha }\\ \; & =-\ln\left(Tr\Omega \right)-\ln(\max_{1\leq i\leq n}\left(\lambda_{i}\right)) \end{array}$$

## Appendix B. Prove the Threshold of Our NH α-Rényi Entropy in ‘Gauge’ Shift

In the following, we will prove that k=2 in the ‘gauge’ shift in Equation (21) is the threshold of the NH entropy trend’s change. For more intuitive equations exhibiting the proof, we investigate the non-normalized matrix Ω of Equation (24), which is the evolution matrix of k=0. We have Tr(Ω):

(A3) $$Tr\left(\Omega \right)=\sum_{i=1}^{3}x_{i}^{2}-2\bar{p}x_{1}x_{2}$$

where $x_{i}$(i=1,2,3), $\bar{p}$ is in the main text, and α is the order in the α-Rényi entropy in Equation (9). Then, we have the following formula of NH α-entropy.

(A4) $$S_{\alpha }\left(\Omega \right)=S_{\alpha }^{H}\left(\rho \right)-\ln\left(\sum_{i=1}^{3}x_{i}^{2}-2\bar{p}x_{1}x_{2}\right)$$

At large time periods, we derive the following:

(A5) $$\lim_{t\to \infty }S_{\alpha }\left(\Omega \right)=-\lim_{t\to \infty }\ln\left(\sum_{i=1}^{3}x_{i}^{2}-2\bar{p}x_{1}x_{2}\right)=-2\mu \tau +\mathrm{constant}$$

where μ, τ, and Δ are in the main text, and the constant is equal to $\left\{\begin{array}{c} \alpha =0,\;2\ln2-\ln\left(1+\frac{1}{\mu^{2}}-\frac{\bar{p}\gamma }{\mu \Delta }\right)\\ \alpha >0,\;\ln2-\ln\left(1+\frac{1}{\mu^{2}}-\frac{\bar{p}\gamma }{\mu \Delta }\right) \end{array}\right..$

When we use parameter *k* by alternating 0, we have the following asymptotical value.

(A6) $$S_{\alpha }{\left(\Omega \right)}_{t\to \infty }=\mathrm{constant}-\lim_{t\to \infty }\ln\left(e^{-k\mu \tau }Tr\left(\Omega \right)\right)=\left(k-2\right)\mu \tau +\mathrm{constant}$$

When k=2, the asymptotical value is a constant. When k>2 (or k<2), the asymptotical value of entropy will approach −∞ (or +∞), and that tendency is proportional to the function with a positive (negative) coefficient. Then, we prove the conclusion with respect to how *k* will impact the asymptotical value in Section 4.
